# Biomechanical simulations of the scoliotic deformation process in the pinealectomized chicken: a preliminary study

**DOI:** 10.1186/1748-7161-2-16

**Published:** 2007-11-09

**Authors:** Pierre Lafortune, Carl-Éric Aubin, Hugo Boulanger, Isabelle Villemure, Keith M Bagnall, Alain Moreau

**Affiliations:** 1 Department of Mechanical Engineering, Ecole Polytechnique, University of Montreal, P.O. Box 6079, Station Centre-ville, Montreal, Quebec, H3C 3A7, Canada; 2 Research Centre, Sainte-Justine University Hospital Centre, University of Montreal, Montreal, Quebec, Canada; 3 Division of Anatomy/Department of Surgery, University of Alberta, Edmonton, Alberta, Canada; 4 Department of Biochemistry, Faculty of Medicine, University of Montreal, Montreal, Quebec, Canada

## Abstract

**Background:**

The basic mechanisms whereby mechanical factors modulate the metabolism of the growing spine remain poorly understood, especially the role of growth adaptation in spinal disorders like in adolescent idiopathic scoliosis (AIS). This paper presents a finite element model (FEM) that was developed to simulate early stages of scoliotic deformities progression using a pinealectomized chicken as animal model.

**Methods:**

The FEM includes basic growth and growth modulation created by the muscle force imbalance. The experimental data were used to adapt a FEM previously developed to simulate the scoliosis deformation process in human. The simulations of the spine deformation process are compared with the results of an experimental study including a group of pinealectomized chickens.

**Results:**

The comparison of the simulation results of the spine deformation process (Cobb angle of 37°) is in agreement with experimental scoliotic deformities of two representative cases (Cobb angle of 41° and 30°). For the vertebral wedging, a good agreement is also observed between the calculated (28°) and the observed (25° – 30°) values.

**Conclusion:**

The proposed biomechanical model presents a novel approach to realistically simulate the scoliotic deformation process in pinealectomized chickens and investigate different parameters influencing the progression of scoliosis.

## Introduction

The etiology of adolescent idiopathic scoliosis (AIS), a three dimensional deformity of the spine and surrounding paravertebral tissues, remains obscure and many researchers have explored different hypotheses ([1], and see [2] for a review). Among them, paravertebral muscle abnormalities (increased electromyographic activity in the muscles of the convex side of the deformed spine [2-5]) have been observed in scoliotic patients. This force imbalance may in fact be a secondary factor in the development of AIS [2].

The Hueter-Volkman concept of 'growth modulation' [6,7] explains, in a phenomenological way, how asymmetrical loading distribution on vertebral epiphyseal growth plate involved in scoliosis can alter the development of vertebrae and promote vertebral wedging. This created deformity is part of a vicious cycle where vertebral asymmetry is generating a spinal curvature, then accentuating the load asymmetrical distribution in the global spine, leading to further asymmetrical growth and so on [8,9].

Finite element models (FEM) that include the mechanical structure of the musculoskeletal system as well as the growth and growth adaptation processes have been already used in humans to simulate the mechanisms underlying the growth modulation of the spine and its associated structures under specific forces [1,10,11]. However, due to the slow progression of the human scoliotic deformities, relevant parameters of these models are relatively complicated to calculate (e.g. material properties, boundary conditions, parameters of the growth process such as the growth rate, the sensitivity of the growth plate to external loads, etc.).

The pinealectomized chicken model has been widely reported in the literature [12-17], especially to test the effects of melatonin in the progression of AIS. Pineal gland removal shortly after hatching induces scoliosis in 45% [12] to 95% [15] of the cases. Although there are some important differences between avian and human osteology (density, shape, growth, etc), scoliosis developed by chicken has spinal deformity and morphological characteristics similar to those seen in AIS. Another interesting issue is that the chicken develops scoliosis within only one month, so the curvature progression pattern is very easy to evaluate.

The main objective of this study was to develop a FEM of the pinealectomized chicken incorporating the main mechanical characteristics of the spine, as well as vertebral growth and growth modulation. Recent studies have reported the difficulty in extrapolating the etiological factors producing AIS in chicken to human beings [18]. The emphasis of this study was therefore not to investigate the pathogenesis but rather the pathomechanisms underlying the progression of scoliosis. The primary objective was to estimate the magnitude of the coronal plane moment that would be expected in the chicken model while the secondary objective was to study the mechanical phenomena that control the spinal deformity progression in the chicken. The FEM of the pinealectomized chicken thus developed provides a means to understand the mechanical parameters associated with the progression of scoliosis, a study that cannot be implemented on human subjects. Knowledge of the growth mechanism from a validated chicken FEM will help to understand the influence of the geometric simplifications implemented in the spine model and will further help to identify the sensitivity of the model parameters to scoliotic progression. This information could then be adapted to a human spine FEM.

## Methods

### Experimental animals and surgical technique

All procedures of this study were reviewed and approved by the institutional committee for care and use of animals of Sainte-Justine University Hospital.

For this study, newly hatched chicken (Mountain Hubbard) were purchased from a local hatchery. The chickens were divided into three distinct groups (pinealectomized, shams and control). The first group, pinealectomized (n = 76), underwent complete removal of the pineal gland according to a protocol described in the literature [19,20]. Basically, a longitudinal incision is made in the scalp of a newly-hatched, anaesthetised chicken followed by a U shaped incision (1 cm width) in the delicate skull around the confluence of sinuses. A 'flap' of skull is then pulled back to reveal the pineal gland which is removed either by forceps or light suction. The flap is replaced and the skin sutured. The second group, shams (n = 20), underwent superficial cranial incision without the ablation of the pineal gland. All surgeries were performed between day three and five after hatching by the same surgeon. In the last group, the controls (n = 25), the chickens did not undergo any surgical procedure. For all three groups, the chicken were immediately introduced into a 12/12 hours light and dark cycle, and kept in constant environmental conditions of 26°C and 70% relative humidity throughout the experimental procedures.

### Finite element model

Finite element models (FEM) provide a means to simulate a natural physiological/biomechanical phenomenon and to test various hypotheses on the simulated model under multiple conditions in order to deduce an approximate solution. In this study, the spinal anatomy of a chicken was approximated by a geometric model comprising of a system of inter-linked mechanical elements. For example, intricate geometry of the vertebrae was modeled using *freeform surfaces *and, ligaments and muscles were represented as multi-linear springs with appropriate material properties. The regions of interest are discretized into small units or elements (e.g. beam, plate or shell type). Articulation between contacting surfaces are exemplified at the element nodes using appropriate boundary/loading conditions/constraints that defined the associated degree-of-freedom thus simulating, as close as possible, the nature of the actual anatomical interaction. The analysis procedure solves a series of model equations at each node from which mechanical parameters (displacements, stresses, strains etc.) within the elements are derived using shape functions and stress-strain relations assumed during the generation of the model equations. FEM analysis therefore enables a systematic understanding of load distribution parameters and injury mechanisms underlying various pathogenesis hypotheses (e.g. spinal deformities, as described in this paper). Additional details specific to finite element analysis in the biomechanics of scoliosis can be found in Aubin [21] and Aubin et al [22].

**Figure 1:**
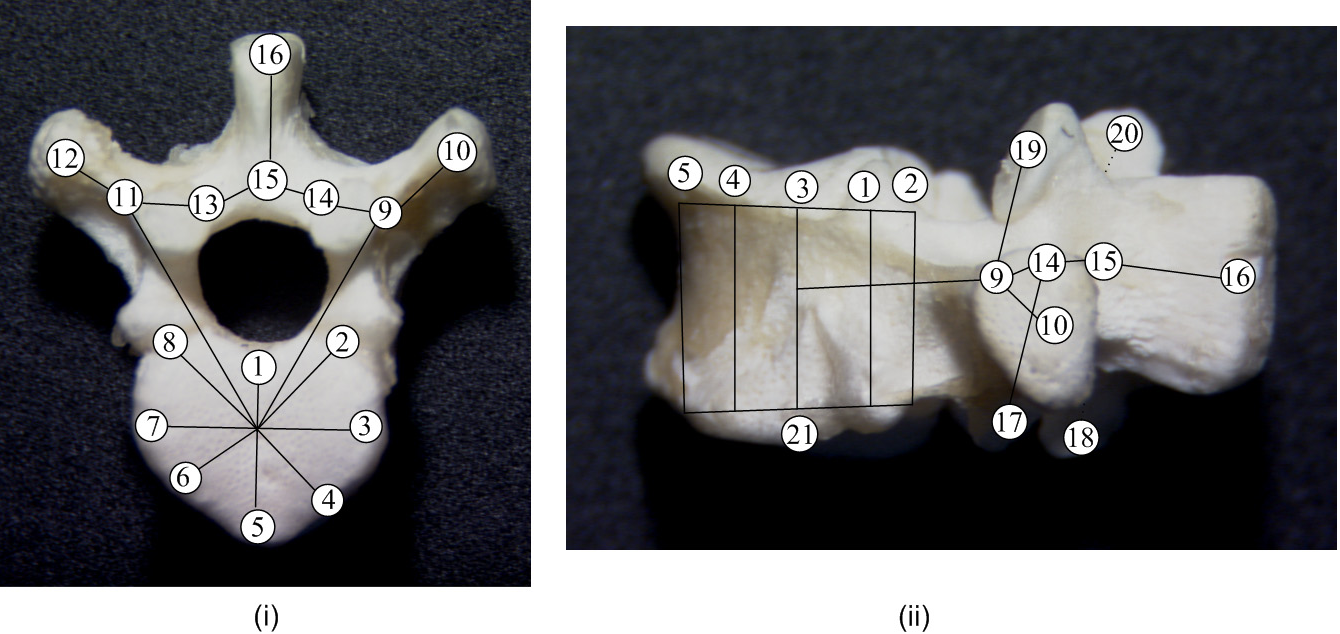
**Position of the landmarks**. Position of the landmarks used to build the FEM: (i) Top view, (ii) lateral view

Creating a FEM model requires information about the material properties of the anatomical entities, their respective surface geometry and the nature of articulation between the anatomical entities in contact. The geometry of each vertebra in the spine of the chicken, from T1 to L1 (8 vertebrae in total), was obtained from 21 anatomical landmarks (12 for the posterior elements and 9 for the vertebral body) measured on one cadaveric vertebra of the control type with a caliper. The measurements were made on the 14th day after hatch (Figure 1 shows the approximate position of the landmarks).

**Figure 2:**
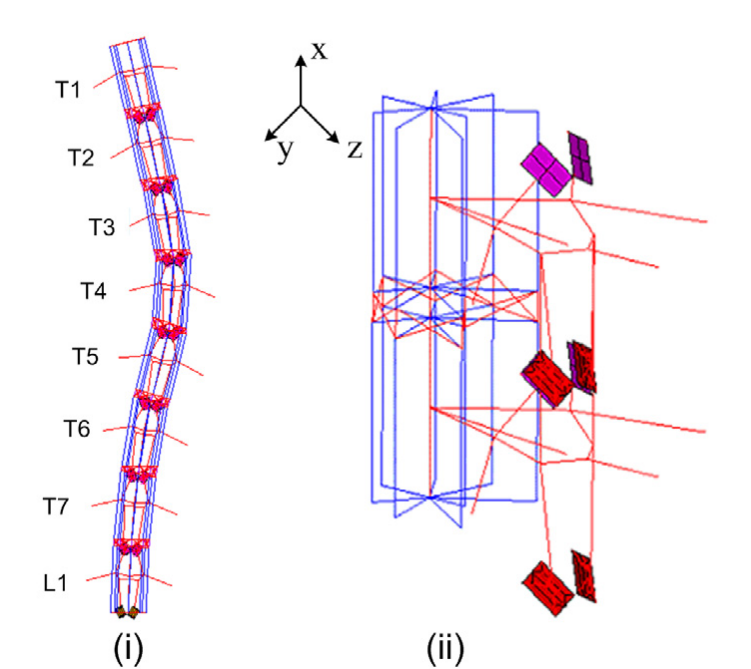
**FEM**. The deformed FEM of the chicken spine: (i) spine in the frontal plane (the 8 vertebrae from T1 to L1); (ii) vertebral motion segment (two vertebrae and one disk).

A finite element model (FEM) previously developed by Villemure et al [1,11] was adapted to the chicken morphology. The original model is a complete representation of the spine, pelvis and thorax used to simulate the scoliosis deformation process in the human spine. It uses a 3D reconstruction technique to recreate the personalized geometry of the patient from calibrated multi-view radiographs. This model has been used thereafter for different projects (effect of brace treatment, spine surgeries, vertebral deformities progression, etc.) Although simplified in terms of geometry, this type of model (beam/link/contact elements) has been proven to be suitable to analyze scoliosis pathomechanisms in humans [11,23,24]. Basically, the mesh and nodes of Villemure's model were adjusted to fit the chicken geometry while respecting the overall topology of the chicken spine (Figure 2).

Each vertebral body was modeled using a system of 3D elastic beams and rigid crossbars (25 elements in total). Local coordinate systems were defined on each vertebral body, with the 'x' axis being perpendicular to the vertebral endplate, and the 'y' and 'z' axis in the plane of the endplate. Eight distributed elements along the vertebral edge enable the evaluation of internal stresses variation within the vertebral bodies and the representation of their wedged shape. The circumferential nodes located on the top of the vertebral body correspond to landmarks 1 to 8 (Figure 1). The middle nodes of the body (one on the upper endplate and one on the lower endplate) are situated approximately in the centre of the body. The height of the body is given by landmarks 21 and 3. Intervertebral disks were modeled similarly (one central beam and eight distributed beams on the circumference), except that tension-only links are inserted in a cross-like fashion to represent collagen fibers (Figure 2). Posterior parts also used 3D beams with landmarks 9 to 20 representing nodes for those elements while the articular facets are represented using shell and contact (point-to-surface) elements. The material properties of human spines were used as an initial approximation (based on experimental data published by Descrimes et al. [25]), as to our knowledge no study provided Young modulus values for the chicken bones or disks. In order to avoid rigid body movements of the overall model, all degrees of freedom at the inferior endplate of L1 were fixed, as well as axial rotation and transverse translations at superior endplate of T1.

### Biomechanical model of bone growth and growth modulation

The growth and its modulation due to mechanical loads were modeled similarly to the phenomenological model proposed by Stokes et al. [24] and adapted by Villemure et al. [1]: the resultant growth included two components. First, the 'normal' endochondral growth of the vertebral body generated at the endplate physes is represented by the baseline growth component *δG*_*x*_. Second, the response of the growth plates to the external loads is modeled by the growth modulation component *δε*_*x*_, where increased compression on the growth plates reduces growth (-*δε*_*x*_), while reduced compression accelerates growth (+*δε*_*x*_), as observed experimentally by Stokes et al. [26]. Both components are perpendicular ('x' direction) to the plane of the growth plates and expressed as a strain increment (mm/mm).

In this model, corresponding deformation increments *δε*_*x *_due to growth modulation is defined by the expression *δε*_*x *_= *β*_*x*_*σ*_*x*_*δG*_*x*_. The relation depends on the baseline growth *δG*_*x*_, on a functional biomechanical stimulus, which corresponds to internal stresses *σ*_*x *_(MPa), and on a parameter *β*_*x *_(MPa^-1^) simulating the sensitivity of bone to that stimulus. In the case of the beam elements used to model the vertebral bodies, the deformation increments *δε*_*x *_due to growth modulation was represented by an equivalent internal modulation force *δF*_*x*_. This force is applied on the nodes of the vertebral bodies lying on the endplates, and is oriented on the axial direction defined by the local coordinate system of the endplate. The force is expressed as follow: *δF*_*x *_= *β *_*x*_(*EF*_*e*_)*δG*_*x*_, where *E *is the Young modulus (MPa) and *F*_*e *_the actual force (N) in the element of the vertebral body directly calculated from the finite element program, as a result of external loading on the vertebrae.

The baseline growth of the vertebral bodies (0.130 mm/day) used in the model was obtained by measurements on the pinealectomized chickens during the simulation period. Growth modulation of the vertebral bodies in 'y' and 'z' directions as well as that in the intervertebral discs were neglected [11]. Although, growth of the posterior parts was neglected, the modulation was indirectly accounted for by dragging the elements with the deformation of the vertebral bodies to maintain coherent geometry of the spine. Initial wedging of the discs was taken into account by the geometric representation of the vertebral endplate anatomy.

### Integration of growth and growth modulation into the FEM

**Figure 3:**
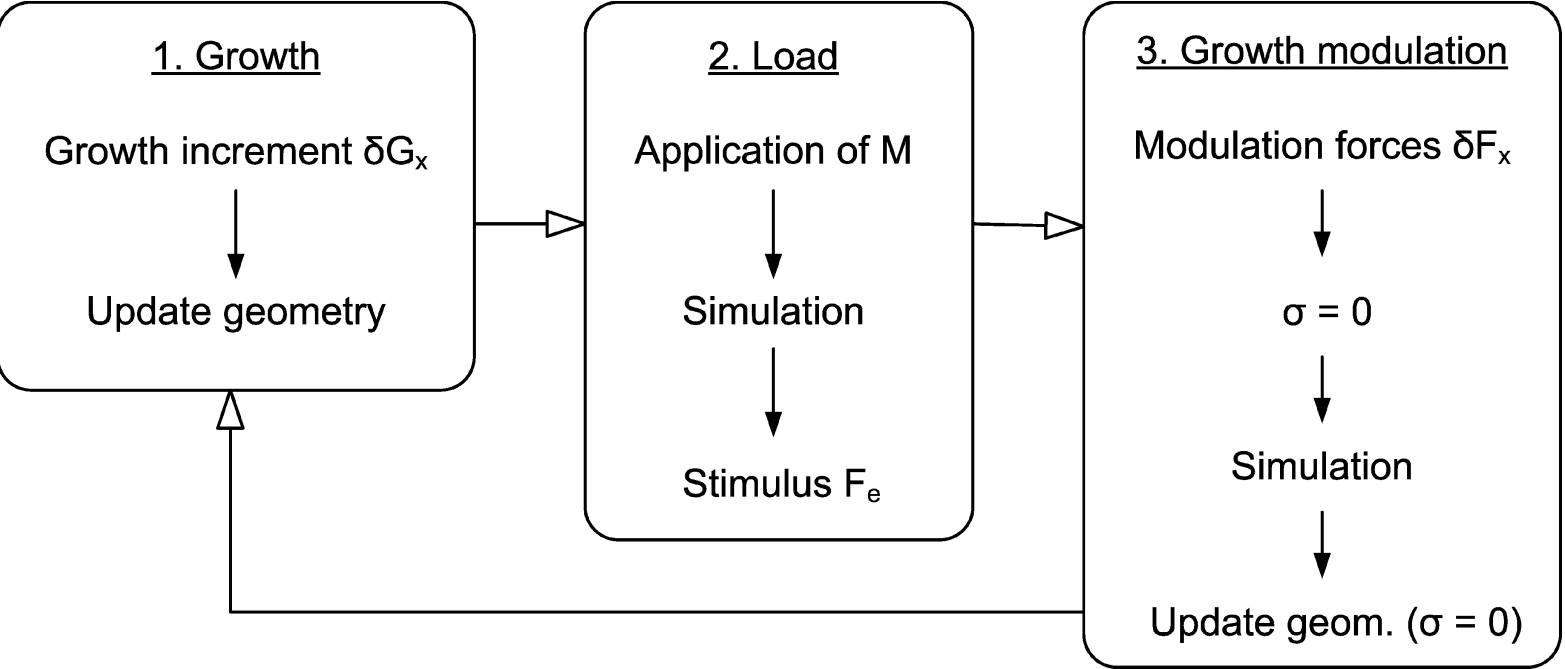
**Flow chart**. Stepwise incremental procedure simulating growth, load and growth modulation.

The entire longitudinal growth of the vertebral bodies was implemented in the finite element software package Ansys 8.0 (Ansys inc., USA) through the following iterative process (Figure 3): *1) Growth*: application of a growth increment and update of the geometry by relocation of nodes; *2) Load*: application of asymmetric loads (a moment representing the asymmetric forces of the muscles at the supposed apex of the curve) and simulation of the FEM, which provides the internal forces *F*_*e *_caused by the moment, as calculated by the finite element software; *3) Growth modulation*: application of the modulated forces *δF*_*x *_calculated with the expression *δF*_*x *_= *β *_*x*_(*EF*_*e*_)*δG*_*x*_, simulation of the FEM and update of the geometry. Note that stresses were set to zero before and after the simulation of the modulated forces in order to perform the step of geometry change due to growth modulation. The accumulation of stresses was indirectly integrated via the geometry, which was modified at each iteration.

### Simulations

Two weeks of growth was simulated based on the observations made on the chicken from the pinealectomized group. The scoliotic deformation appeared early at the second week around the 14^th ^day after hatching. During the next 3 or 4 weeks, scoliosis progressed with a significant deformation. Around the fifth week, spontaneous fusion occurred from the second to the sixth thoracic vertebrae, forming a bony plate [27], as is usually observed in normal chicken. Because we were not interested to represent this phenomenon which does not occur in the human spine, simulations were conducted over a period of 2 weeks in the 14–28 days after hatching period.

In a scoliotic subject, the normal pre-stress of the discs [28] is affected by the unequal loads of the musculo-ligamentous structures [29]. An unpublished in-house study, performed on pinealectomized chickens, investigated muscle recruitment patterns associated with walking, by recording EMG activation in different paraspinal muscles. Aside from a decrease in bone mass density after pinealectomy, the findings also revealed an increase in muscle tone during rest and an asymmetry in EMG activation patterns during walking. To simulate this complex force pattern, a range of asymmetric loads (2–14 Nmm) were applied at the supposed apical vertebra (T5) to produce bending in the frontal plane of the spine. These loads correspond to a range of possible moments caused by suspected muscle activity imbalance. The applied moment therefore creates a shift from the normal load on the vertebrae (stabilizing action of muscles), and consequently produces an asymmetric growth plate activity. As mentioned above, this unbalance accentuates the spinal curvature according to the Hueter-Volkman concept. The load values were chosen to create a coherent deformation of the spine and were considered to be plausible taking into account the size of the animal. The incremental procedure of Figure 3 was repeated over 14 cycles, each cycle representing a day. The value of the parameter *β *was set to 1.87 MPa^-1^, as obtained empirically by Stokes [30]. More recent data are available [31], but since a variation of this parameter allows simulating modulations of different intensity the present value was found to be adequate for this study. A future objective however is to investigate the influence and the validity of this complex parameter. The solution considered non-linearity due to large displacements and change of state at the contact elements of the articular facets. At each iteration, three geometric descriptors were evaluated. The Cobb angle of the spine is defined as the angle between the perpendiculars at the inflexion points of the spinal curve in the frontal plane. Wedging angle of the apical vertebra is the angle between the endplates of the most off-centered vertebra in the frontal plane. Finally, the lateral displacement of the vertebral body is the distance between the deformed position of one vertebra and its original position.

## Results

### Experimental results

**Figure 4:**
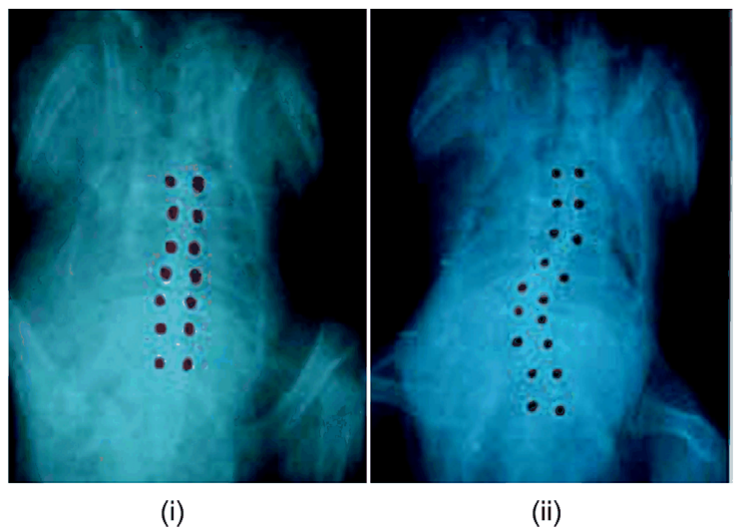
**Radiographs**. Radiographs of a sham chicken (i) and of a pinealectomized chicken at day 28 (ii).

None of the *shams *or *control *chicken developed scoliosis. Within the first group (*pinealectomized*), 55% of the animals (n = 42) developed a scoliosis. Chickens underwent radiological analysis on day 28 to measure the scoliotic deformities (Figure 4). The Cobb angle values were quite variable, with a mean value of 26° and a standard deviation of 12° (range between 5° to 56°). Few chickens developed a double curve. In those situations, only the most severe deformation was considered. Because of the low level of definition on the radiographs, the wedging angle of individual vertebrae was measured only when the vertebra corners were visible. The most deformed vertebrae were generally located near the apex of the curvature. A typical wedge angle was around 25° to 30° for a severe scoliosis after four weeks.

### Numerical results

**Figure 5:**
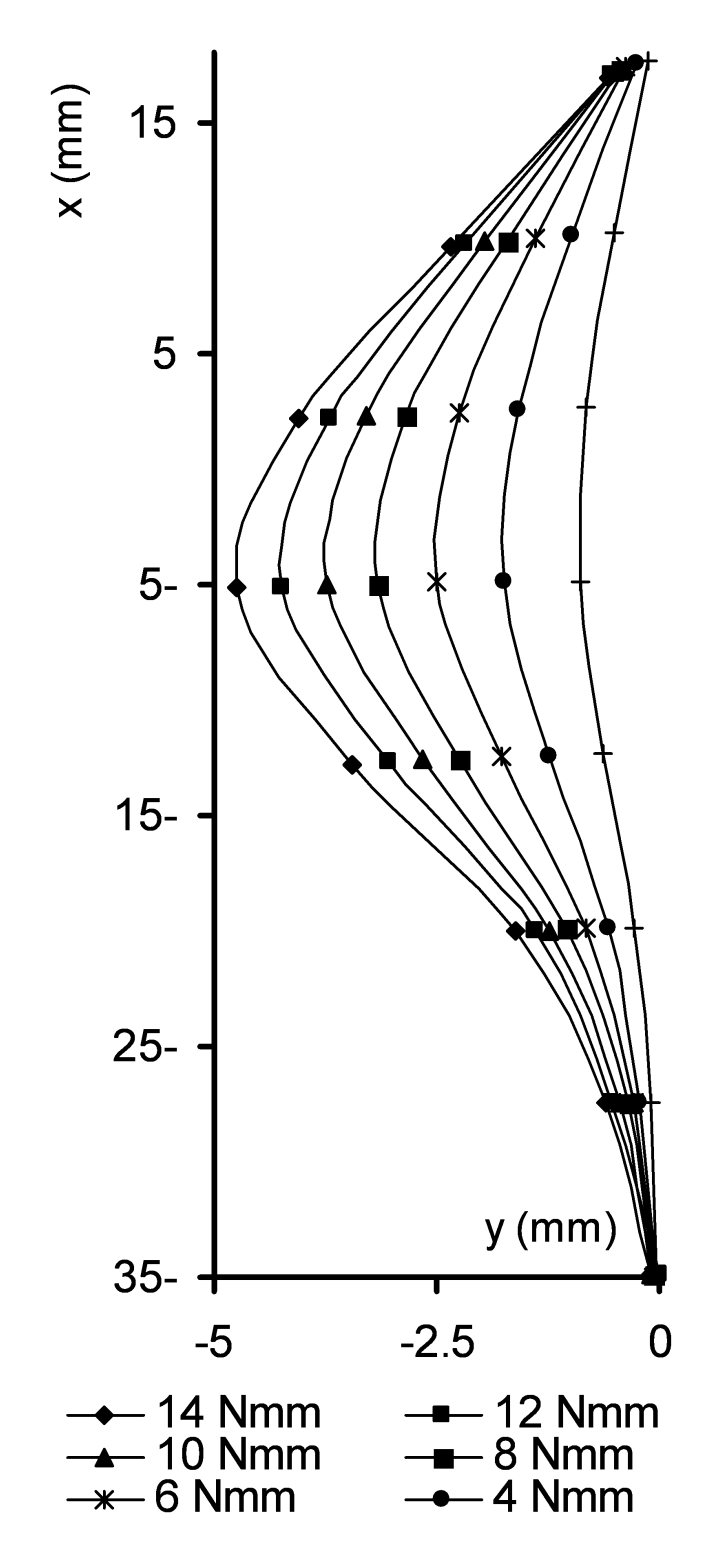
**Simulated curves**. The Simulated spinal curves (vertebral body centroids), for moments of 14, 12, 10, 8, 6, 4 and 2 Nmm.

By varying the value of the applied moment, different scoliosis configurations were simulated. The resulting Cobb angle varied between 6° and 37°. The maximal vertebral wedging (range between 5° to 28°) appeared at T4 for moments of 14, 12 and 10 N.mm, and at T5 for the other applied moments. Lateral displacement of the apical vertebrae varied from 1 to 5 mm. The segment of the spine studied had an initial length of 39 mm, while final length was around 53 mm after the application of the moments and growth processes. Table 1 summarizes the scoliotic descriptors for the seven loading cases simulated, and Figure 5 shows the lateral displacements of the vertebral bodies.

**Table 1:** Numerical simulation results

Applied moment (Nmm)	Cobb Angle (degrees)	Wedging angle (Max.) (degrees)	Latteral displacement (Max.) (mm)
14	37	28	4.7
12	32	25	4.2
10	27	22	3.7
8	22	18	3.2
6	17	14	2.5
4	12	10	1.7
2	6	5	0.9

Numerical simulation results of the deformed spine after 14 days of growth, for the different applied moments. Cobb angle, wedging angle and lateral displacements are in the frontal plane.

### Preliminary validation

**Figure 6:**
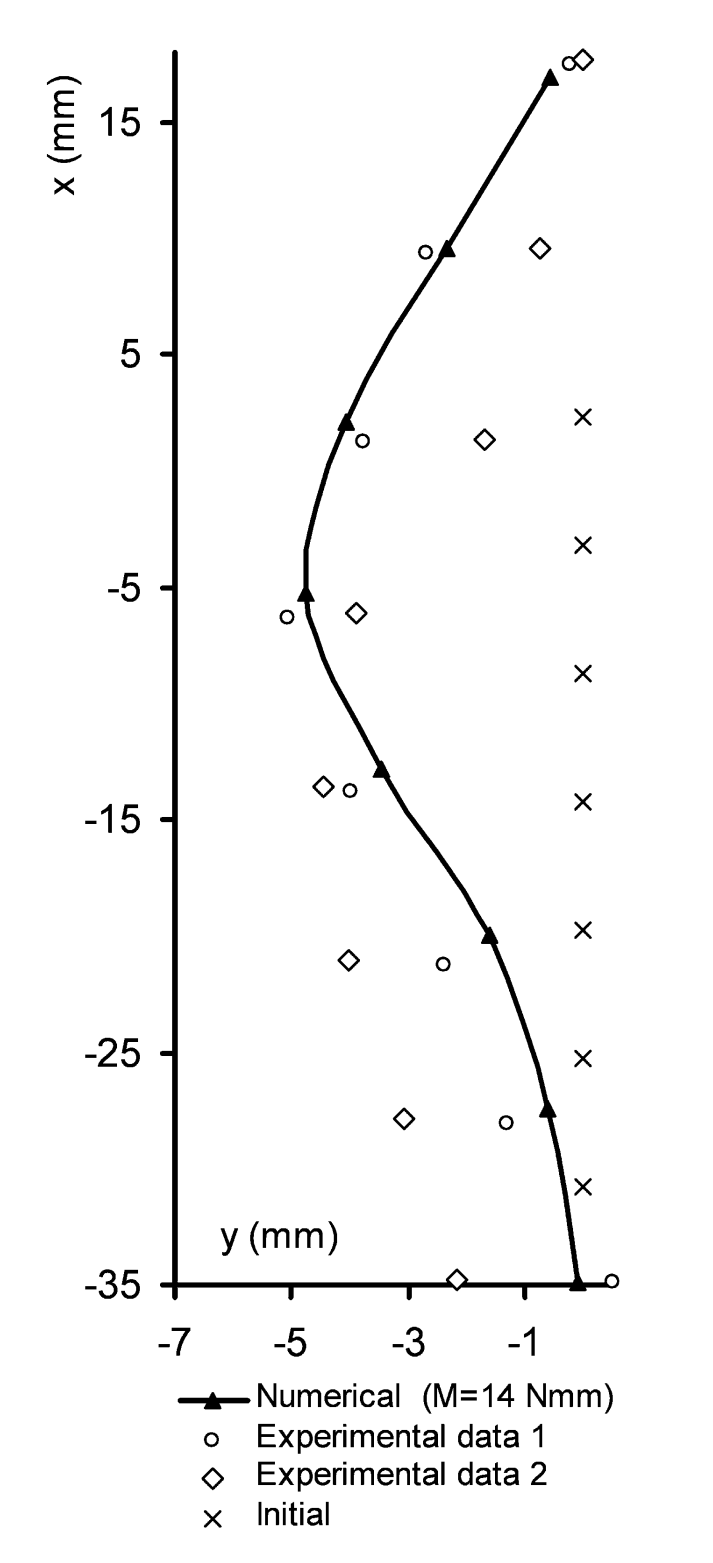
**Comparison of simulations and clinical curves**. Position in the frontal plane of the vertebral bodies for two selected curves and for the simulation (moment values of 14 Nmm). The initial position of the vertebrae is also illustrated.

A descriptive comparison of the simulation results with the experimental deformation patterns was made as a preliminary validation. Among the 42 pinealectomized chickens that developed a scoliosis during the experimental study, two of them have been chosen for that purpose. Chickens produce a large variety of deformations, so we are reporting here the results of two cases that showed a single curve, a moderate to important Cobb angle, and an apex located approximately at the middle of the spine. These were considered as typical cases most representative of the single thoracic spine deformity. Figure 6 illustrates a comparison of the numerical and the experimental deformations in the frontal plane obtained when a moment of 14 N.mm was applied.

## Discussion

The deformed shape simulated was similar to the one seen in the scoliotic chickens. The Cobb angle produced by the numerical simulation was 37°, while the ones observed on the selected chickens were 41° and 30°. Apical vertebrae of the model and the two experimental spines were situated at T4. The growth of the spine was slightly underestimated in the numerical simulations due to the fact that we neglected the growth of the discs. Finally, the vertebral wedging value of 28° reached similar values than the experimental values (25° – 30°). The relation between the applied moment and the scoliotic descriptors presented a non-linear pattern, due to the non-linear behavior of the growth introduced by the contact elements representing the articular facets.

Other experimental studies have shown similar results. Inoh et al. [32] obtained an average Cobb angle of 12.9° on a group of 22 pinealectomized chickens at the age of 4 weeks. Turgut et al. [17], with a group of 6 chickens of 8 weeks old, obtained an average Cobb angle of 18°. Machida et al. [15] studied separately wedged-shape and non-wedged-shape deformities on chickens aged from 1 to 20 weeks. They obtained average Cobb angle of 59° and 26° for the wedged-shape and the non-wedged-shape groups. Cheung et al. [33] reported wedging in the thoraco-lumbar junction, with the apex at either T7 or L1, and not in the thoracic region. Those results present different experimental conditions, and are not directly comparable with our results, but they show that the simulated Cobb angles (6° to 37°) are in the same order of magnitude. Even if all those studies reported the vertebral wedged near the apex, none of them gave an angle value for this deformation. However, approximate measurements made from their published radiographs of cadaver chickens were showing wedge angles in the range of 30° – 40°, which is comparable with the numerical values (25° – 30°) obtained in this study.

Some factors are limiting the use of the chicken model to study AIS. Indeed, the fusion occurring in the thoracic spine, limiting the evaluations over a period of three weeks, is a main obstacle and difference with the human spine. Very few data are available on the growth parameters and materials properties of the chicken. In particular, parameter *β*_*x*_, simulating the sensitivity of bone to the growth stimulus (*σ*_*x*_), is very difficult to evaluate, and may be different between the specimens. At present, there are no studies that have documented spinal loading imbalance and EMG patterns in animal subjects. Research should be pursued in this direction in order to better understand the spinal forces imbalance. Finally, the horizontal (versus vertical in human) position of the thoracic and lumbosacral spine changes the way that gravity forces act on the vertebral growth plates, which perhaps limits the role of gravity loads in the progression cycle of chicken scoliosis, as compared to the effects of gravity on the whole human spine.

The growth process of the scoliotic spine was monitored under different asymmetric spinal loading conditions wherein the loads were selected from a range of plausible values. It is to be noted that the simulation model provided a quantitative representation of the growth process in the scoliotic spine and its prediction of curve evolution is only valid within the bounds of the estimated parameters of the model. However, the qualitative interpretation of the results is limited due to the estimations made in the model. Furthermore, since the objective of the study was to examine the pathomechanics of scoliosis we cannot comment on the cause/effect relationship between asymmetric spinal loading and scoliosis.

## Conclusion

This paper presents an adaptation of a previously developed FEM used here to develop a novel approach to study scoliosis. The agreement between the experimental study and the simulations showed the feasibility of the biomechanical model to realistically simulate the scoliotic deformation process in pinealectomized chickens and investigate different parameters influencing the progression of scoliosis.

When further developed and completely validated, this modeling approach could help investigating the influence and the sensitivity of additional parameters on the progression of scoliosis, such as materials properties, sensitivity factor of bone to mechanical stimulus, boundaries conditions, etc. A few studies have reported similar anatomical characteristics in the scoliotic development induced by pinealectomy in chickens to those of human idiopathic scoliosis [34-36]. The challenge now is to use this new tool and to eventually find ways to transfer the knowledge to understanding that pathomechanics underlying the progression of idiopathic scoliosis in humans.

## Competing interests

The author(s) declare that they have no competing interests.

## Authors' contributions

PL participated in the concept and design of the study, carried out the computer simulations and drafts the manuscript. CEA and IV participated in the coordination, concept and design of the study and helped to draft the manuscript. HB performed the experimental data acquisition and analysis and helped to draft the manuscript. KMB and AM performed the experimental data acquisition and analysis and participated in coordinating the study.
